# Nasal oral fistula revealing Langerhans´ cell histiocytosis in adult patient: case report

**DOI:** 10.11604/pamj.2021.40.16.27074

**Published:** 2021-09-06

**Authors:** Anis Mzabi, Maissa Thabet, Taghrid Tlili, Hend Zorgati, Jihed Anoun, Imen Ben Hassine, Monia Karmeni, Fatma Ben Fredj, Moncef Mokni, Chadia Laouani

**Affiliations:** 1 Internal Medicine Department, Sahloul Hospital, Faculty of Medicine Sousse, University of Sousse, 4000 Sousse, Tunisia,; 2 Pathology Department, Farhat Hached Hospital, Faculty of Medicine Sousse, University of Sousse, 4000 Sousse, Tunisia

**Keywords:** Langerhans cell histiocytosis, adult, histiocytofibroma, chemotherapy, case report

## Abstract

Langerhans cell histiocytosis (LCH) is a rare systemic disease caused by proliferation of mature histiocytes; its association to histiocyto fibroma is rarely reported. It rarely affects adults. We report a case of systemic LCH, in an adult patient with osteolytic lesion causing a fistula between the left nasal cavity and hard palate, involving the bone, lung, lymph node and associated to multiple histiocyto fibroma. The patient was operating for a fistula, and he was treated by chemotherapy and corticosteroids. Langerhans´ cell histiocytosis is a rare case, especially in adult patient. The diagnosis was based on histological and immunohistochemical analyses. This patient was treated by steroids and chemotherapy.

## Introduction

Paul Langerhans Jr. first described Langerhans´ cell histiocytosis (LCH) in 1868 [1]. Histopathologically, it is generally defined by CD1a+/Langerin+ Langerhans-like cells. The specific origin of LCH cells has not yet been identified. The annual incidence of LCH in children aged less than 15 years is around 5 to 9/106 and 1/106 in patients older than 15 years of age [2]. This disease may affect one organ or more, considered as a multisystem disorder. Bone and skin are the most involved. Diabetes insipidus (DI) is the most common initial sign of central nervous system (CNS) involvement. Isolated pulmonary LCH (PLCH) is a specific type of LCH and it is the most common manifestation in adult patients [3]. This form is strongly related to smoking and smoking cessation could lead to remission without treatment. Here, we report a case of LCH that showed the involvement of multiple organs including bone, lung, lymph node and testicular with atypical cutaneous lesions, in a 39-year-old man and revealed by a nasal-oral fistula.

## Patient and observation

**Patient information:** it is about a 39-year-old man with 20 pack years of smoking and a past history of extraction of multiple teeth and placement of a dental prosthesis. The extraction sites showed prolonged or incomplete healing. The patient presented a swallowing disorder related to a fistula between the left nasal cavity and hard palate. Indeed, he reported a history of a deterioration of the general condition with asthenia and weight loss of 7kg in 2 months without polydipsia or polyuria.

**Figure 1:**
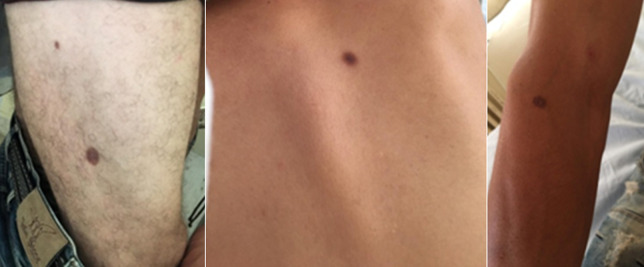
skin examination- purpuric nodules on the back, thigh and forearm with a size of 2cm suggesting multiple histiocytofibroma

**Figure 2:**
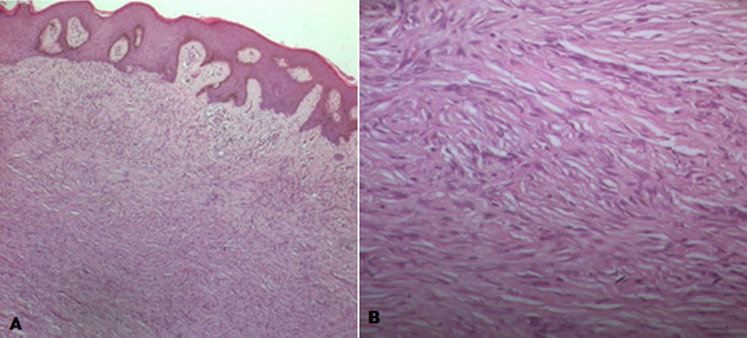
skin biopsy- histiocyto fibroma (dermatocyto fibroma): the reticular dermis has a non-encapsulated lesion of moderate cell density made up of spindle cells; the epidermis is acanthotic and has hyperpigmentation; (A: original magnification HEx25); (B: original magnification HEx400)

**Figure 3:**
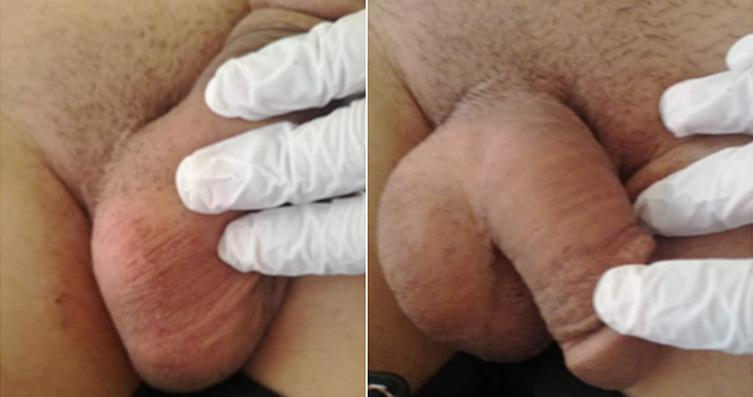
external genitalia varicocela

**Clinical findings:** physical examination revealed nasal oral communication through a fistula between the nasal cavity and the hard palate with the presence of left carotid jugular and supra clavicular and inguinal lymphadenopathy. Skin examination revealed the presence of purpuric nodules on the back, thigh and forearm with a size of 2cm which appeared a few months ago (Figure 1), and in the biopsy of a skin lesion in the thigh histological appearance of a dermatofibroma (histiocytofibroma) (Figure 2). Examination of the external genitalia revealed normal-sized testes with bilateral hydrocele (Figure 3) confirmed by ultrasound. The rest of the physical examination is unremarkable.

**Figure 4:**
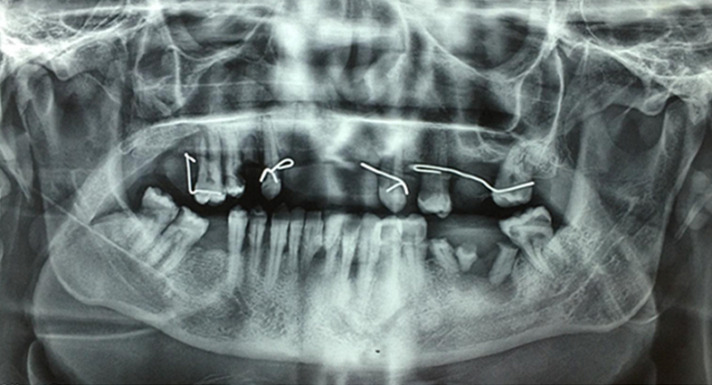
panoramic X-ray showing generalized aggressive perodontitis

Diagnostic assessment: Laboratory investigations revealed that the patient´s erythrocyte sedimentation rate (ESR) was 10mm/1h (≤ 15mm/1h), the Creactive protein (CRP) was 12mg/L. The blood urea nitrogen 3mmol/L, and creatinine 58 µmol/. The liver function was normal (ALT/ AST (UI/l): 17/14). Syphilis, hepatitis and HIV serology were negative. Complete blood count (CBC) showed that white blood cells (WBC) count was at, lymphopenia at 700 element/L, haemoglobin level was 11g/dl, and platelets were at 155000. TSH was at 1.24mUI/L. The panoramic X-ray showed aggressive periodontitis (Figure 4).

**Figure 5:**
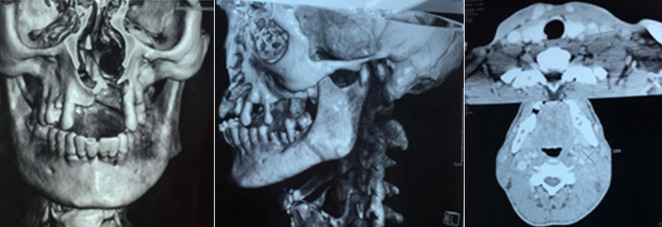
computed tomography scan of the facial mass showing a fistula between the left nasal cavity and hard palate

**Figure 6:**
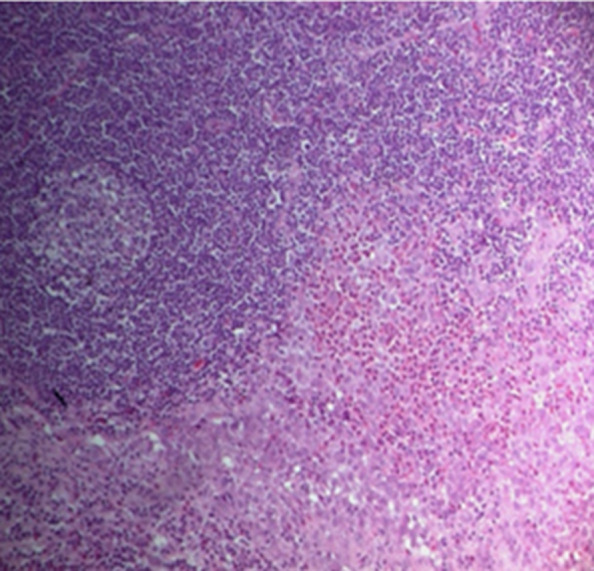
a lymph node biopsy- Langerhans Cell Histiocytosis (LCH) (original magnification HEx100)

**Figure 7:**
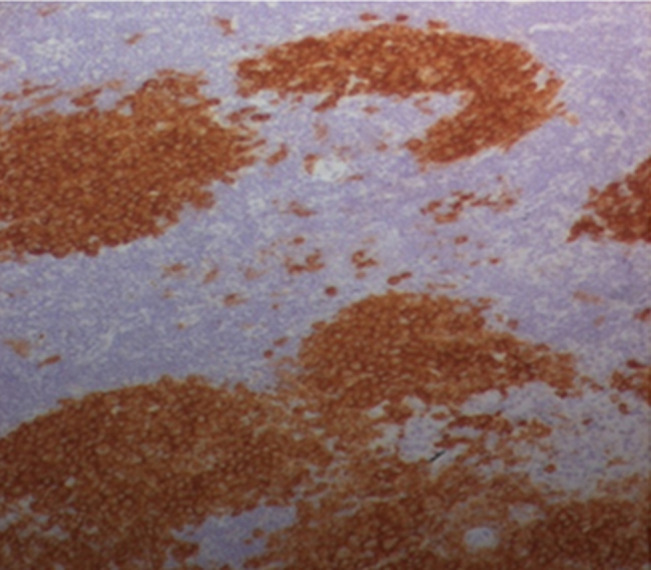
Langerhans Cell Histiocytosis (LCH) (Immunohistochemical stain for Langerhans cell-specific CD1a antigen)

The CT scan of the facial mass highlighted a large bucco-nasal communication measuring 1.5cm with a dehiscence of the bony palate opposite the implantation site of the upper incisors (11 and 12) with widening of the desmodental space around the root of 28 rarefaction of the alveolar bone and condensation of the underlying basal bone (Figure 5). A nasal endoscopy with a biopsy of the cavum returned without abnormality. The biopsy of the palatal revealed a moderate non-specific chronic inflammatory reaction. A lymph node biopsy was performed and confirmed the Langerhans Cell Histiocytosis (LCH) based on the morphological (Figure 6) and the immunohistochemical examination which showed that histioid cell cytoplasm and nucleus were positive for CD1a and SP100 marker (Figure 7). The diagnosis of LCH has been made and the extension assessment showed bilateral pulmonary cysts and nodules of infracentimetric size, a bone destructive osteolytic lesion of the vertebral bodies of the L2, D10 and D1, bilateral inguinal and latero-aortic sub-renal lymphadenopathy and Left retrocolic tissue nodule. At the colonoscopy: presence of multiple plane polyps and the biopsy was normal. The dental examination of our patient revealed generalized aggressive generalized periodontitis.

**Therapeutic intervention:** based on the above results, the diagnosis of Langerhans cell histiocytosis (HandSchuller-Christian Disease) was validated.

**Follow-up and outcomes:** the patient was operating for a fistula, and he was treated by vinblastine and corticosteroids.

## Discussion

LCH is a rare proliferative disorder with 3 clinical variants respectively: eosinophilic granuloma; Hand-Schuller-Christian disease and Letterer-Siwe disease.

Any bone can be involved, but in the majority of cases, LCH involves the skull, rib, spine, and long bone. Clinically, LCH usually presents as swelling or pain. Although lytic bony lesions are typically appearing, LCH could show a more aggressive pattern of osteolysis. Remodelling of the bone may cause an expanded appearance, as in this case.

Maxillary or mandibular involvements seem particularly common in adults. It is often a source of diagnostic errors and significant sequelae with dental loss [4]. Pulmonary LCH presents as multiple nodules and cysts on high-resolution CT, and these lesions generally affect the upper and middle lung zones with sparing of lung bases as in our case.

Literature on oral LCH region is limited and mostly consists of case reports or retrospective series [5, 6]. In some cases, surgical treatment has been shown to be very effective in the treatment of localized oral manifestations of LCH and is sometimes combined with steroid injections [6]. Radiation therapy may serve as an alternative or second line treatment in case of impossible surgical removal. Digestive involvement is exceptional in adults. A series shows that at this age, it is most often a single colonic lesion (polyp, ulceration) discovery by chance, without extra-digestive manifestation [7].

The skin involvement in LCH is polymorphic. It could be a singular lesion or, more often, multi-focal involvement. It can appear as scaly papules or squamous-crusted, sometimes seborrheic or eczema-like in appearance or brown/red nodules sometimes ulcerated. They mainly affect the trunk, face and scalp. The major folds of the intertrigo type are frequent and may take on an erosive aspect, including perineal. The perianal or vulvar locations are not rare in adults and can be very disabling. The injuries of the oral mucosa are usually associated with underlying bones involvement.

Our patient presented histocytofibroma which is rarely associated to histiocytosis. Two cases have been reported [8]. Although LCH is not fatal in all cases, delayed diagnosis or treatment can lead to serious impairment of organ function and decreased quality of life. To our days, there are no standard therapies and no prospective trials for adult LCH. Treatment is based on LCH organ involvement and the extent of the disease [3]. Local therapies are recommended for isolated skin or bone involvement. Surgical excision of the lesion can be undertaken in very limited cases of SS-LCH [3]. Systemic therapy is strongly recommended for MS-LCH or SS-LCH with multi-focal bone lesions. No standard treatments have been identified but a combination of vinblastine and prednisolone therapy could be the standard option, as in pediatric LCH.

Adult patients who undergo vinblastine + prednisolone treatment tend to have higher toxicity than children [9]. Our patient received vinblastine and prednisolone with good outcomes. In summary, we described a case of LCH involving the bone, lung, lymph node, with a difficulty to discriminate LCH from other disorders. Therefore, systemic evaluation is needed when LCH is suspected with biopsy of any suspicious lesion.

## Conclusion

In summary, we described a case of LCH involving the bone, lung, lymph node, with a difficulty to discriminate LCH from other disorders. Therefore, systemic evaluation is needed when LCH is suspected with biopsy of any suspicious lesion.
